# Increased Serum Levels of Proinflammatory Cytokines Are Accompanied by Fatigue in Military T-6A Texan II Instructor Pilots

**DOI:** 10.3389/fphys.2022.876750

**Published:** 2022-04-28

**Authors:** Elizabeth G. Damato, Seth J. Fillioe, Seunghee P. Margevicius, Ryan S. Mayes, Jonathan E. Somogyi, Ian S. Vannix, Alireza Abdollahifar, Anthony M. Turner, Lidia S. Ilcus, Michael J. Decker

**Affiliations:** ^1^ Department of Physiology and Biophysics, School of Medicine, Case Western Reserve University, Cleveland, OH, United States; ^2^ Naval Medical Research Unit Dayton, Dayton, OH, United States; ^3^ Frances Payne Bolton School of Nursing, Case Western Reserve University, Cleveland, OH, United States; ^4^ Department of Population and Quantitative Health Sciences, School of Medicine, Case Western Reserve University, Cleveland, OH, United States; ^5^ 711th Human Performance Wing, U. S. Air Force School of Aerospace Medicine, Dayton, OH, United States; ^6^ United States Air Force, Washington, DC, United States

**Keywords:** fatigue, cytokines, inflammation, hypergravity, hyperoxia, military aviator

## Abstract

Tactical aviation imposes unprecedented physical challenges including repetitive exposure to hypergravity, hyperoxia, increased work of breathing, and profound cognitive workloads. Each stressor evokes outcomes ranging from musculoskeletal duress and atelectasis to physical and cognitive fatigue, the latter among the foremost threats to aviators. Whereas sleep loss is traditionally considered the primary cause of fatigue in aviators, converging experimental, observational, and medical studies have identified biochemical mechanisms promoting onset of fatigue. Those mechanisms, which fundamentally differ from sleep loss, revolve around increased proinflammatory cytokines, produced and released in response to tissue injury, chronic inflammatory disorders, allergens, or physical duress. This study’s objective was to inform our understanding of potential relationships between serum levels of proinflammatory cytokines and onset of fatigue within a cohort of aviators who experience multiple high-performance sorties on a daily basis.

**Methods:** Active duty and reservist T-6A Texan II instructor pilots were studied on three separate days across their week-long flying schedule. Data collected included a physical assessment, subjective fatigue levels, venous blood samples for measures of chemistry and serum analytes, and urine samples for specific gravity.

**Results:** Twenty-three persons were studied, of which 22 fulfilled minimum study requirements of completing two sorties. The study cohort was comprised of primarily males, age 37.95 ± 4.73 years with a BMI of 26.63 ± 3.15 kg/m^2^. Of 37 measurable serum analytes, 20 differed significantly (*p* < 0.05) between baseline values with those measured at the study endpoint. Thirteen of the aviators reported increased fatigue scores across their flying schedule whereas nine did not. Eleven blood serum analytes were associated with increasing levels of fatigue.

**Discussion:** Fatigue in aviators has been attributed almost solely to sleep loss, nocturnal sorties, or disrupted circadian rhythmicity. In contrast, our study findings suggest an alternative mechanism that can promote onset of fatigue: increased blood levels of proinflammatory cytokines. Specific mechanisms triggering synthesis and release of those cytokines and other analytes are yet to be determined. However, their expression patterns suggest responses to both chronic and acute inflammation, hyperoxia, or bronchopulmonary responses to inspiration of dry gas, positive airway pressure, or perhaps atelectasis.

## Introduction

Of the many challenges posed by the aviation environment, fatigue is among the foremost threats to aviators. The National Commission of Aviation Safety asserts that the “pervasive sense of burnout and chronic fatigue that exists throughout the military aviation enterprise is contributing to unsafe conditions” (Cody et al., 2020). The Commission predicts that fatigue-related degradation to human performance will be the “likely cause of the next mishap” (Cody et al., 2020). Their conclusions echo studies confirming that fatigue contributed to 4–8% of all aviation mishaps (Caldwell, 2005) and has accounted for 13–25% of Class A mishaps (damage of $2 million or more) with fatalities (Gaines et al., 2020).

Fatigue can have an insidious onset that renders an individual unaware of their deteriorating physical and cognitive performance (Bourgeois-Bougrine et al., 2003; Gibson et al., 2003). Additional challenges are imposed when considering whether those decrements are due to fatigue (Decker et al., 2009) or sleepiness, which is prevalent in our society (Decker, 2010). While standardized definitions distinguish fatigue from sleepiness, (Hossain et al., 2005; Matthews and Hancock, 2017; Hu and Lodewijks, 2020), not all disciplines have adopted them (Hu and Lodewijks, 2020). Human performance specialists define fatigue as “a disabling symptom in which physical and cognitive function is limited by interactions between performance fatigability and perceived fatigability” (Enoka and Duchateau, 2016). Performance fatigue is further defined as diminished force production capacity of a muscle group (Gandevia, 2001); cognitive fatigue is the reduction in cognitive performance that emerges following mental exertion (Boksem and Tops, 2008). Those definitions are contrasted by The International Civil Aviation Organization (ICAO), which defines fatigue as “A physiological state of reduced mental or physical performance capability resulting from sleep loss or extended wakefulness, circadian phase, or workload (mental and/or physical activity) that can impair a crew member’s alertness and ability to safely operate an aircraft or perform safety-related duties” (ICAO, 2013).

Sleep loss is considered the primary cause of fatigue in aviators (Caldwell et al., 2009; Gore et al., 2010; Caldwell et al., 2019; Morris et al., 2020; Wilson et al., 2020; Wingelaar-Jagt et al., 2021). However, multiple experimental, observational, and medical studies have identified biochemical mechanisms promoting the onset and maintenance of fatigue as well as sleepiness (Raison et al., 2006; Raison et al., 2009; Dantzer et al., 2014). Those biochemical mechanisms, which differ fundamentally from sleep loss, center around immune system activation followed by release of both pro- and anti-inflammatory cytokines (Dantzer et al., 2014; Karshikoff et al., 2017). Functions of those signaling molecules range from activating the body’s defense systems against invading pathogens to initiating tissue repair from trauma (Cai et al., 2021), rheumatic disorders (Korte and Straub, 2019), or physical duress (Philippou et al., 2009). Although interrelated, fatigue and sleepiness represent two separate threats to aviator safety.

The biochemical activities of proinflammatory cytokines are not constrained to the site of injury. Upon entering the systemic circulation, they travel throughout the neurovascular system, increasing permeability of the blood brain barrier (Dantzer et al., 2014). After traversing that barrier, proinflammatory cytokines accumulate within the neural extracellular milieu, eventually achieving neurotoxic levels (Dantzer et al., 2014). This triggers microglial cells to begin restorative processes. Their activity reduces bioavailability of amino acids and neurotransmitter precursors, leading to diminished synaptic levels of key monoamines that sustain cortical arousal: norepinephrine, dopamine and serotonin (Dantzer et al., 2014). Functional outcomes include cortical hypoarousal which manifests as cognitive fatigue, reduced working memory, distractibility, lack of motivation and eventually, depressive behaviors (Decker et al., 2005; Blier and Briley, 2011; Dantzer et al., 2014).

Increased systemic and central nervous system levels of proinflammatory cytokines are established biochemical promoters of both fatigue and sleepiness (Raison et al., 2010; Dantzer et al., 2014; Sofroniew, 2014; Karshikoff et al., 2017; Zielinski et al., 2019; Lasselin et al., 2020), two primary threats to aviator safety. However, it has been unclear whether tactical aviators experience increased levels of proinflammatory cytokines with concomitant fatigue. This knowledge gap poses a barrier to defining causes of aviator fatigue other than sleep loss (Caldwell et al., 2019). To inform our understanding of the relationship between inflammatory processes with fatigue in aviators, we characterized blood serum proinflammatory cytokine and fatigue levels, blood chemistry and other physiologic measures within a cohort of T-6A Texan II instructor pilots. We hypothesized that exposure to the physical challenges of performance aviation (sorties), including musculoskeletal duress (Wagstaff et al., 2012; Kollock et al., 2016; O'Conor et al., 2020), high gravitational accelerations (+Gz) (Grossman et al., 2012), hyperoxia (Damato et al., 2020), increased work of breathing (West, 2013), and acceleration atelectasis (West, 2013; Pollock et al., 2021), could lead to synthesis and release of proinflammatory cytokines (Dantzer et al., 2014). We also speculated that recurrent exposure to those physical challenges, occurring over several consecutive days would promote a continual increase of both proinflammatory cytokine levels and fatigue.

## Methods

**FIGURE 1:**
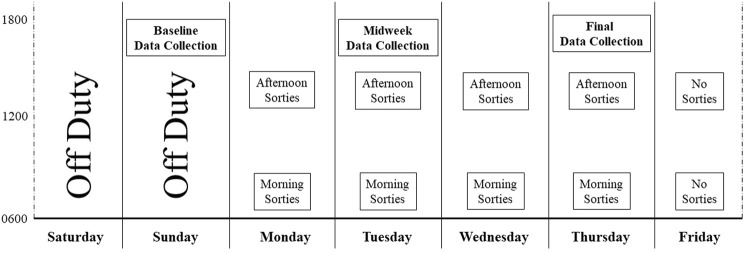
Study data collection overview. This figure provides a graphical overview of data collection time with the timing of sorties occurring over the week-long study period. Data obtained at each of the three collection points is detailed in Figure 2.

**FIGURE 2:**
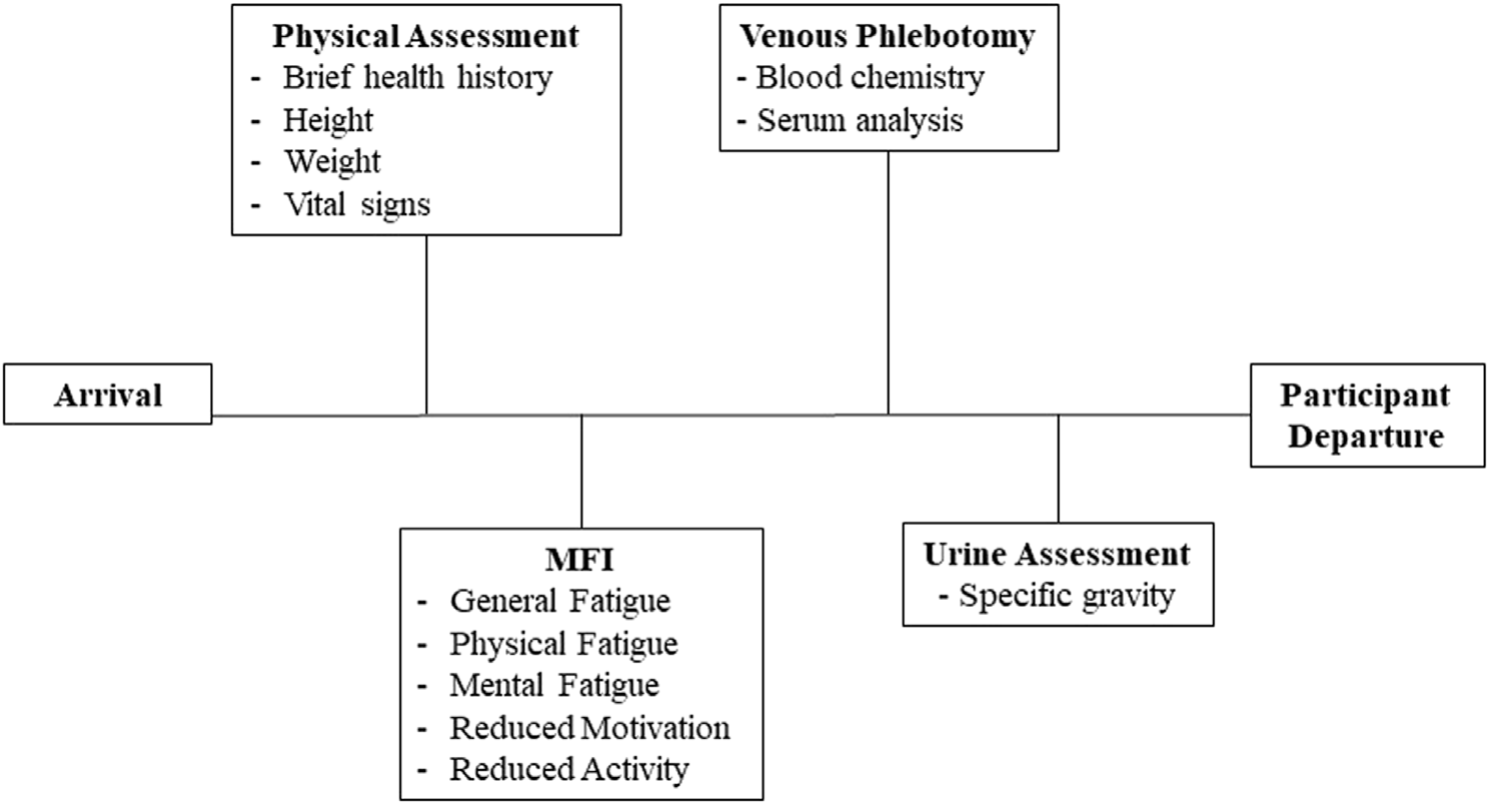
Biologic and self-reported data. This figure illustrates the specific biological samples and self-reported data collected during baseline (Sunday), midweek (Tuesday) and the study endpoint (Thursday) at each station. Data collection required approximately 40 min and only occurred after each participant’s completion of their scheduled activities.

The study protocol was approved in advance by the Naval Medical Research Unit-Dayton Institutional Review Board (protocol # NAMRUD. 2020.0004); a Reliance agreement was arranged with Case Western Reserve University (STUDY20210542). All potential study participants were required to be military aviators and scheduled to fly at least two sorties during the week of data collection, in aircraft capable of high gravitational acceleration that also delivered continuous oxygen-enriched gas mixtures through a mask. Candidate participants received a verbal description of the study protocol and purpose. Of those, 24 persons provided written informed consent to participate, and 23 were studied (one withdrew prior to the start of data collection). Data were collected on three separate days across the week-long flying schedule: Sunday, Tuesday, and Thursday (Figure 1). No flying occurred on Friday of the study week. Data collected at each time point included a physical assessment, subjective fatigue levels, venous blood for measures of blood chemistry and serum analytes, and a urine sample for measurement of specific gravity (Figure 2).

### Physical Assessment

Each participant was assessed for a history of prior or current fatiguing illness (e.g. Epstein Barr), current medications, along with measurement of height, weight, body temperature, and vital signs. Participants provided a single urine sample of approximately 50 ml. A 1.0 ml sample was pipetted onto a refractometer (Tekcoplus RETK-70, Kowloon, Hong Kong) to measure the urine specific gravity.

### Measures of Fatigue

The Multidimensional Fatigue Inventory (MFI) was used to assess five dimensions of fatigue: Physical Fatigue, Mental Fatigue, General Fatigue, Reduced Activity, and Reduced Motivation. The MFI is self-administered and requires approximately 5 min to answer 20 Likert-style statements with a scale of responses ranging 1–5. Higher scores for each dimension correspond with higher levels of fatigue (Lin et al., 2009).

### Blood Sample Collection

Blood sampling was performed as followed: a tourniquet was applied approximately 2–3 inches above the right or left antecubital vein. A 23-gauge butterfly needle with tubing was used to puncture the vein. An 8.5 ml BD Vacutainer^®^ serum separation tube (tiger top) was attached to the butterfly tubing, followed by collection of ∼4 ml of venous blood. The Vacutainer tube was centrifuged at 3000 rpm for 8–10 min. The separated serum was pipetted into a total of three 1.5 ml Eppendorf tubes, which were immediately placed in dry ice and stored at -70°C until analyses. All blood sampling was performed at the end of the workday, and therefore, a non-fasting condition.

Upon removal of the vacutainer from the butterfly tubing, a 1.0 ml heparinized syringe was attached to the tubing and an additional venous sample collected. That sample was immediately injected into an iSTAT blood analyzer (Abbott, Abbott Park, IL) equipped with a CHEM8+ cartridge for analyses of pH, Sodium (Na), Potassium, (K), Chlorine (Cl), Ionized Calcium (iCa), Anion Gap (AnGap) and Total Carbon Dioxide (TCO_2_), Glucose (Glu), Blood Urea Nitrogen (BUN), and Creatinine. The CHEM8+ also measured percent Hematocrit (Hct) and from that calculated the hemoglobin concentration (Hb). Lactate was measured using the Lactate Plus Blood Lactate meter (Nova Medical, Waltham, MA).

### Serum Measurements of Cytokines, Chemokines and Angiogenic Proteins

Serum was analyzed with multi-array technology (QuickPlex SQ; Meso Scale Diagnostics, Rockville, MD) using the V-PLEX neuroinflammation panel (Catalog No. K15210D) and Obesity panel (Catalog No. K15277K). Each panel contained a series of preconfigured 96-well plates, with each well containing carbon electrodes prepared with a specific capture-antibody. Serum was placed into each well where analytes of interest were captured by the antibody-coated carbon electrodes and then detected by an analyte-specific ruthenium-conjugated secondary antibody. When the secondary antibody was electrochemically stimulated, it emitted light with the intensity determined by the concentration of the analyte within the sample (Chowdhury et al., 2009). Analyte concentrations were quantified with an 8-point calibration curve that was constructed from light signal intensities generated from a series of known concentration standards.

Analysis of each participant’s serum sample, collected at each of the three study time points, included dividing each sample between two adjacent wells of each 96-well plate to establish measurement reproducibility. Serum levels of each analyte of interest were measured using the following 96-well plates:

Proinflammatory plate measuring interleukin 1 beta (IL-1β), interleukin 2 (IL-2), interleukin 4 (IL-4), interleukin 6 (IL-6), interleukin 8 (IL-8), interleukin 10 (IL-10), interleukin 13 (IL-13), tumor necrosis factor alpha (TNF-α), and interferon-gamma (IFN-γ).

Chemokine plate measuring monocyte chemoattractant protein-1 (MCP-1), monocyte chemoattractant protein-4 (MCP-4), eotaxin, macrophage inflammatory protein-1 alpha (MIP-1α), eotaxin-3, thymus activation regulated chemokine (TARC), macrophage inflammatory protein-1 beta (MIP-1β), macrophage-derived chemokine (MDC), and interferon-gamma inducible protein of 10 kDa (IP-10).

Cytokine plate measuring interleukin 1 alpha (IL-1α), interleukin 5 (IL-5), interleukin 7 (IL-7), interleukin 12 (IL-12), interleukin 15 (IL-15), interleukin 16 (IL-16), interleukin 17A (IL-17A), tumor necrosis factor beta (TNF-β), and vascular endothelial growth factor A (VEGF-A).

Angiogenesis plate measuring basic fibroblastic growth factor (bFGF), vascular endothelial growth factor C (VEGF-C), vascular endothelial growth factor D (VEGF-D), vascular endothelial growth factor receptor 1 (Flt-1), placental growth factor (PlGF), and tyrosine kinase 2 (Tie-2).

Vascular Injury plate measuring serum amyloid A (SAA), C-reactive protein (CRP), intercellular adhesion molecule-1 (ICAM-1), and vascular cell adhesion molecule-1 (VCAM-1).

Obesity plate measuring brain-derived neurotrophic factor (BDNF), ghrelin, leptin, fibroblast growth factor 21 (FGF-21), and glucagon.

Together, these plates enabled measurements of 42 analytes within each serum sample obtained at each of the three blood collection time points. This totaled 126 serum analyte measurements per participant over the 1-week study period.

### General Statistical Approaches

Sample size estimation and power analyses were based on a minimum statistical power of 0.85 at a two-tailed significance of *p* < 0.05 using data generated from our preliminary studies of pre and post sortie blood serum measurements of analyte expression. In the current study, each serum analyte was measured at three separate time points (Sunday-baseline, Tuesday-midweek, and Thursday-endpoint). Analyte measures were continuous in nature and descriptive analyses with normality plots provided information of data distributions. The change in General Fatigue scores at study endpoint from baseline was dichotomized into two groups: Increase in Fatigue vs. No Increase in Fatigue. A repeated measures linear mixed modelling approach with compound symmetric covariance structure was used to assess the overall effect of time, with pairwise comparisons between baseline and each time point. This robust approach compensates for unbalanced groups and allows flexibility for selection of covariance structures as well as relaxation of sphericity assumptions. Comparisons of fatigue scores were also tested using a repeated linear mixed model including group by time interaction with compound symmetric covariance structure. Exploratory analyses were conducted between blood serum analyte levels with MFI scores. Those analyses employed a General Linear Model (GLM) adjusted for baseline values of serum biomarkers in which participants with no increase in General Fatigue scores were considered as the “reference group.” If analyte values were not normally distributed, data were transformed using natural logarithm prior to the analysis. Bonferroni-Holm corrections were applied for controlling Type I error.

We measured and compared the following variables: 1) five domains of fatigue measured with the MFI, 2) venous blood chemistry, 3) 42 blood serum analytes, 4) urine for measurement of specific gravity, 5) venous blood lactate levels, and 6) number and duration of sorties flown, and highest hypergravity (G force) level reached during each sortie. Values are presented as the mean ± one SD and range, unless otherwise specified. All tests were two-sided and *p*-values less than 0.05 were considered as statistically significant. Data were analyzed using Statistical Analysis Software (SAS) version 9.4 (SAS Institute, Cary, NC) and Statistical Package for Social Sciences (SPSS) version 28 (IBM Corporation, Armonk, NY).

## Results

Twenty-three persons were studied of which 22 fulfilled minimum study requirements of completing two sorties during the data collection period. Data presented here represent the study cohort (N = 22) comprised of primarily males, age 37.95 ± 4.73 years with a BMI of 26.63 ± 3.15 kg/m^2^ (Table 1). All study participants denied a history of fatigue-inducing pathology, such as Epstein Barr virus (EBV), cytomegalovirus (CMV), or severe acute respiratory syndrome coronavirus 2 (SARS-CoV2). No participant was febrile at any time during the study period and measures of blood pressure were within normal limits.

**TABLE 1:** Study participant demographics. There were no age or BMI differences between males and females (*p*-values from Wilcoxon rank sum test).

Sex	Age (years) M ± SD (Range)	BMI (kg/m^2^) M ± SD (Range)
Males *n* = 20	37.95 ± 4.73 (29–47)	26.63 ± 3.15 (21.92–32.63)
Females *n* = 2	41.00 ± 0.00 (41–41)	24.38 ± 1.61 (23.24–25.52)
*p*-value	0.21	0.36

Urine specific gravity and blood lactate values were within the normative clinical range (Antinone and Kress, 2009; Brescon et al., 2018) at each data collection time point, without significant changes occurring from baseline to final (Tables 2, 3). Venous blood chemistry values also showed no changes from baseline to final (Table 3).

**TABLE 2:** Hydration status. Calculated plasma volumes during each study time point were within the range of euhydration (2.98–3.19 L) (Yiengst and Shock, 1962). Baseline urine specific gravity suggested mild dehydration (defined as > 1.020) whereas midweek and study endpoints were within the range of euhydration to hypohydration (≥1.013 to ≤1.020) (Brescon et al., 2018).

	Baseline M ± SD (Range)	Midweek M ± SD (Range)	Final M ± SD (Range)	*p*-value Baseline to final
Calculated Plasma Volume (L)	3.11 ± 0.36 (2.37–3.90)	3.06 ± 0.34 (2.37–3.90)	3.07 ± 0.32 (2.37–3.69)	0.155
Urine Specific Gravity^a^	1.021 ± 0.009 (1.002–1.036)	1.020 ± 0.009 (1.004–1.040)	1.017 ± 0.008 (1.004–1.032)	0.162

a n = 21 due to one missing urine sample at baseline. All *p*-values are from repeated measures linear mixed model with compound symmetric covariance structure analysis.

**TABLE 3:** Blood chemistry. Venous blood chemistry measurements reveal no change in the levels of 12 analytes measured at baseline, midweek, and study endpoint. All *p*-values are from repeated measures linear mixed model with compound symmetric covariance structure analysis.

Analyte	Baseline M ± SD (Range)	Midweek M ± SD (Range)	Final M ± SD (Range)	*p*-value Baseline to final
Sodium (Na)	140.09 ± 1.72 (135–142)	140.14 ± 1.61 (137–143)	140.32 ± 1.32 (138–144)	0.612
Potassium (K)	3.84 ± 0.29 (3.4–4.6)	3.95 ± 0.22 (3.5–4.4)	3.89 ± 0.16 (3.7–4.2)	0.504
Chloride (Cl)	103.00 ± 2.43 (99–109)	102.00 ± 2.31 (98–107)	102.23 ± 2.49 (98–109)	0.084
Ionized Calcium (iCa)	1.27 ± 0.04 (1.2–1.36)	1.27 ± 0.03 (1.22–1.36)	1.27 ± 0.03 (1.22–1.33)	0.804
Total Carbon Dioxide (TCO_2_)	25.09 ± 1.97 (22–29)	25.32 ± 1.81 (23–28)	25.45 ± 1.95 (22–30)	0.275
Glucose (Glu)	95.00 ± 11.16 (75–125)	98.55 ± 14.69 (85–145)	98.68 ± 14.61 (85–134)	0.269
Blood Urea Nitrogen (BUN)	19.23 ± 6.43 (7–34)	17.73 ± 3.88 (8–27)	17.09 ± 4.37 (9–27)	0.064
Creatinine (Crea)	1.10 ± 0.25 (0.6–1.8)	1.01 ± 0.21 (0.7–1.4)	1.02 ± 0.19 (0.7–1.5)	0.109
Hematocrit (Hct)	42.91 ± 3.28 (35–48)	43.82 ± 2.02 (41–47)	43.50 ± 2.63 (38–48)	0.200
Hemoglobin (Hgb)	14.59 ± 1.11 (11.9–16.3)	14.89 ± 0.68 (13.9–16.0)	14.79 ± 0.90 (12.9–16.3)	0.191
Anion Gap	16.82 ± 2.22 (9–19)	17.77 ± 1.02 (16–20)	17.59 ± 0.96 (15–19)	0.112
Lactate	1.09 ± 0.36 (0.5–2.0)	0.89 ± 0.32 (0.5–1.7)	1.01 ± 0.49 (0.5–2.8)	0.345

Fatigue levels of the entire cohort in each of the five MFI domains measured at each data collection time point are presented in Table 4. Baseline fatigue levels were comparable to those reported by persons defined as “well” during a population-based study of fatigue (Lin et al., 2009). General Fatigue scores increased across the study period, becoming significantly greater than baseline. The General Fatigue scores during each data collection time point ranged between 4 and 14. As scores >10 have been associated with a state of being “unwell” (Lin et al., 2009), some study participants may have also experienced corollary symptoms of “unwellness” that were not captured during our assessments.

**TABLE 4:** Multidimensional Fatigue Inventory. Group scores for the entire study cohort for each of the five domains measured at baseline, midweek and study endpoint are presented in vertical columns. By study endpoint, scores for General Fatigue were greater than baseline. All *p*-values are from repeated measures linear mixed model with compound symmetric covariance structure analysis.

MFI dimension	Baseline M ± SD (Range)	Midweek M ± SD (Range)	Final M ± SD (Range)	*p*-value Baseline to final
Physical Fatigue	6.23 ± 2.27 (4–12)	6.36 ± 2.11 (4–10)	6.59 ± 2.06 (4–10)	0.165
Mental Fatigue	7.23 ± 2.31 (4–12)	7.41 ± 2.30 (4–12)	7.45 ± 2.15 (4–13)	0.618
General Fatigue	7.91 ± 2.89 (4–15)	8.82 ± 2.59 (4–13)	9.23 ± 3.13 (4–14)	0.008^a^
Reduced Activity	6.09 ± 1.87 (4–10)	6.00 ± 2.02 (4–10)	5.91 ± 1.85 (4–11)	0.375
Reduced Motivation	6.32 ± 1.49 (4–10)	6.00 ± 1.90 (4–10)	6.27 ± 2.49 (4–9)	0.893

a Indicates significance following Bonferroni-Holm correction for multiple comparisons.

The increase in General Fatigue levels that emerged across the study prompted us to explore whether it occurred in all study participants. Participants were then categorized into two groups; those whose final General Fatigue scores were higher than baseline (*n* = 13) and those whose final General Fatigue scores were not higher than baseline (*n* = 9). Table 5 displays baseline, midweek, and endpoint MFI scores for all five MFI dimensions within those two groups. A repeated measures linear mixed model with compound symmetric covariance structure analysis including fatigue group by time interaction was performed to compare values at each of the three data collection time points. The MFI scores did not differ between the two groups at baseline or midweek. However, by the study endpoint, participants reporting increased General Fatigue also endorsed increased levels of Mental Fatigue, Reduced Motivation, and Reduced Activity, although only Reduced Motivation remained significant following Bonferroni-Holm correction.

**TABLE 5:** Comparisons of Multidimensional Fatigue Inventory Scores between participants with and without Increased General Fatigue. This table provides comparisons between MFI scores of participants categorized according to whether their General Fatigue scores at the study endpoint were increased from baseline (*n* = 13) or not increased from baseline (*n* = 9). Participants with increased General Fatigue also reported increased levels of Mental Fatigue, Reduced Motivation, and Reduced Activity at the study endpoint, although only Reduced Motivation remained significant following Bonferroni-Holm correction. All *p*-values are from repeated measures linear mixed model with compound symmetric covariance structure analysis including fatigue group by time interaction.

MFI scores	Increased n = 13 M ± SD (Range)	Not increased n = 9 M ± SD (Range)	*p-*value
Physical			
Baseline	6.38 ± 2.50 (4–12)	6.00 ± 2.00 (4–9)	0.435
Midweek	6.46 ± 2.03 (4–9)	6.22 ± 2.33 (4–10)	0.403
Final	7.15 ± 2.08 (4–10)	5.78 ± 1.86 (4–9)	0.202
Mental			
Baseline	7.00 ± 2.04 (4–10)	7.56 ± 2.74 (4–12)	0.117
Midweek	6.92 ± 2.10 (4–12)	8.11 ± 2.52 (4–12)	0.596
Final	7.92 ± 2.18 (4–13)	6.78 ± 2.05 (4–10)	0.036
General			
Baseline	7.77 ± 2.80 (4–13)	8.11 ± 3.18 (4–15)	0.770
Midweek	9.38 ± 2.22 (6–13)	8.00 ± 3.00 (4–13)	0.239
Final	10.85 ± 2.41 (7–14)	6.89 ± 2.57 (4–12)	0.002^a^
Reduced Activity			
Baseline	6.62 ± 1.98 (4–10)	5.33 ± 1.50 (4–8)	0.929
Midweek	6.69 ± 2.21 (4–10)	5.00 ± 1.22 (4–7)	0.329
Final	6.62 ± 1.94 (4–9)	4.89 ± 1.17 (4–7)	0.045
Reduced Motivation			
Baseline	6.69 ± 1.49 (4–9)	5.78 ± 1.39 (4–8)	0.479
Midweek	6.54 ± 2.11 (4–11)	5.22 ± 1.30 (4–8)	0.261
Final	7.23 ± 2.68 (4–14)	4.89 ± 1.36 (4–8)	0.007^a^

a Indicates significance following Bonferroni-Holm correction for multiple comparisons.

Each study participant provided a self-report of their number of daily sorties, the duration of each sortie and maximal +Gz obtained. From this, we calculated the average number of sorties completed by participants in the study cohort (*n* = 22) to be 4.68 ± 1.43 and the total time spent in sorties was 412 ± 126.05 min. We then determined that aviators who experienced increased General Fatigue and Reduced Motivation (*n* = 13) completed 5.15 ± 1.28 sorties and their total time spent executing those sorties was 449.15 ± 93.68 min. Aviators who did not report increased General Fatigue (*n* = 9) completed 4.00 ± 1.41 sorties and their cumulative time spent executing those sorties was 358.33 ± 151.86 min. Biserial correlation analyses yielded significant positive correlations between study participants with increased General Fatigue and their number of completed sorties (r = 0.50, *p* = 0.017) and total time spent executing sorties (r = 0.45, *p* = 0.036). In addition, Spearman correlation analysis suggested that maximum +Gz was significantly associated with increased General Fatigue scores at the study endpoint (Thursday) (r = 0.59; *p* = 0.008).

Serum levels of 42 analytes were measured in venous blood samples obtained at each of the three data collection time points. Of those 42 analytes, five were below the limits of detection: IL-1β, IL-2, IL-1α, IL-5, TNF-β. Of the remaining 37 analytes, 20 differed significantly between baseline values (obtained Sunday) with those measured at endpoint (Thursday), coinciding with the end of the week’s flying schedule. Serum levels for all 20 analytes and adjustment of significance following Bonferroni-Holm correction are provided in Table 6. This table reveals that IL-4 and IL-8, both measured by the proinflammatory plate, increased between baseline and endpoint data collection, whereas IL-10 decreased. The chemokine plate reported that MCP-1, MCP-4, Eotaxin-3, TARC, MIP-1α and MIP-1β were increased. The cytokine plate revealed increased levels of IL-7, IL-12, IL-15, IL-17A and VEGF-A. The Angiogenic and Vascular plates demonstrated that Tie-2, VCAM-1 and VEGF-D were reduced. The obesity panel revealed BDNF was increased while both leptin and glucagon were reduced.

**TABLE 6:** Serum analyte levels. Twenty analytes changed significantly between baseline and endpoint; 13 remained different following Bonferroni-Holm correction.

	Baseline M ± SD (Range)	Midweek M ± SD (Range)	Final M ± SD (Range)	p-value Baseline to final
Proinflammatory Plate (pg/ml)				
IL-4	0.05 ± 0.06 (0.005–0.26)	0.05 ± 0.07 (0.003–0.25)	0.07 ± 0.05 (0.02–0.22)	0.001^a^
IL-8	8.46 ± 3.63 (4.38–16.42)	10.37 ± 5.01 (4.24–20.64)	11.00 ± 3.92 (5.99–20.76)	0.003^a^
IL-10	0.41 ± 0.36 (0.12–1.59)	0.38 ± 0.35 (0.09–1.59)	0.26 ± 0.20 (0.09–1.03)	0.018
Chemokine Plate (pg/ml)				
MCP-1	127.42 ± 45.02 (55.21–267.11)	162.81 ± 68.35 (59.19–311.9)	175.33 ± 70.61 (55.83–336.39)	0.002^a^
^b^MCP-4	41.86 ± 37.65 (9.80–184.13)	50.57 ± 35.05 (7.13–163.06)	54.97 ± 34.24 (16.14–163.30)	0.008
^b^Eotaxin-3	6.75 ± 5.36 (1.40–22.88)	10.32 ± 6.29 (0.24–23.79)	13.44 ± 7.17 (3.06–35.44)	0.002^a^
TARC	73.58 ± 54.21 (16.50–230.36)	101.55 ± 70.12 (23.27–293.91)	110.64 ± 50.26 (21.51–229.57)	0.009
^b^MIP-1α	8.99 ± 4.65 (3.61–24.27)	11.55 ± 9.16 (2.21–41.03)	10.41 ± 2.76 (4.08–15.39)	0.020
^b^MIP-1β	68.77 ± 23.70 (37.91–136.96)	82.79 ± 37.19 (36.00–167.29)	88.60 ± 28.10 (45.13–159.98)	0.002^a^
Cytokine Plate (pg/ml)				
IL-7	10.90 ± 5.81 (3.80–27.46)	14.90 ± 7.81 (4.04–36.02)	20.28 ± 8.11 (7.40–34.56)	<0.0001^a^
^b^IL-12	71.88 ± 31.33 (36.46–173.69)	74.22 ± 39.84 (27.36–191.47)	89.59 ± 42.94 (39.91–237.60)	0.0001^a^
IL-15	2.06 ± 0.30 (1.66–2.61)	1.94 ± 0.46 (1.12–2.72)	2.27 ± 0.34 (1.76–3.04)	0.018
IL-17A	1.09 ± 0.35 (0.42–1.83)	1.24 ± 0.60 (0.45–2.72)	1.74 ± 0.52 (1.11–3.34)	<0.0001^a^
^b^VEGF-A	43.25 ± 27.34 (8.58–134.42)	70.46 ± 63.03 (9.27–263.24)	118.28 ± 63.05 (34.82–276.96)	<0.0001^a^
Angiogenesis Plate (pg/ml)				
^b^VEGF-D	1551.86 ± 405.37 (1068.68–2709.09)	1357.24 ± 388.18 (834.71–2523.37)	1148.91 ± 286.80 (762.09–1961.71)	**<**0.0001^a^
^b^Tie-2	980.50 ± 235.84 (274.87–1363.72)	900.91 ± 253.11 (296.72–1355.75)	737.84 ± 175.32 (187.54–1046.33)	**<**0.0001^a^
Vascular Panel Plate (pg/ml)				
^b^VCAM-1	640629.35 ± 293150.58 (322327.73–1261489.42)	517489.62 ± 215525.34 (238663.56–1067055.15)	465626.35 ± 103173.38 (326028.23–741180.39)	0.006^a^
Obesity Panel Plate (pg/ml)				
^b^BDNF	17.72 ± 23.86 (3.88–106.87)	171.51 ± 222.87 (4.30–673.10)	372.95 ± 443.62 (9.35–1727.51)	<0.0001^a^
^b^Leptin	7579.36 ± 6122.32 (398.99–24226.97)	7207.24 ± 5036.27 (634.99–16857.55)	6247.65 ± 5414.35 (384.04–17973.04)	0.015
^b^Glucagon	24.57 ± 11.04 (6.14–48.84)	13.88 ± 9.57 (2.29–43.04)	10.83 ± 9.09 (2.74–45.02)	<0.0001^a^

a Indicates significance following Bonferroni-Holm correction for multiple comparisons.

b Indicates natural logarithm transformation prior to analysis using repeated measures linear mixed model with compound symmetric covariance structure.

Table 7 presents outcomes from the GLM analyses between all blood serum analytes with levels of General Fatigue. Eleven blood serum analytes were associated with increasing levels of General Fatigue. These included MCP-1, MCP-4, eotaxin-3, TARC, MIP-1β, IL-15, VEGF-A and BDNF, which were previously presented in Table 6 due to their significant changes across the study period. During GLM analyses, three additional analytes emerged as being associated with General Fatigue: TNFα, eotaxin and MDC.

**TABLE 7:** Association between mean change in serum analytes with General Fatigue Scores. General Linear Modelling (GLM) identified 11 blood serum analytes that were associated with increased levels of General Fatigue. Estimate **β** of the General Fatigue Score (column 2) utilizes participants who did not report an increase of General Fatigue between baseline and the study endpoint as the reference group.

Analyte	Estimate (β) of General Fatigue score	SE	95% CI	*p*-value Baseline to Final^a^
Proinflammatory				
^b^TNF α	0.1620	0.0745	(0.0059, 0.3180)	0.0427
Chemokine				
MCP1	95.7499	21.2057	(51.3659, 140.1340)	0.0002^c^
^b^MCP4	0.6250	0.1459	(0.3196, 0.9305)	0.0004^c^
Eotaxin	55.3390	21.2115	(10.9429, 99.7352)	0.0173
^b^Eotaxin-3	0.5525	0.2455	(0.0387, 1.0662)	0.0364
TARC	49.9432	17.3052	(13.7229, 86.1634)	0.0095
MIP-1β	29.4478	9.7810	(8.9759, 49.9197)	0.0072
^b^MDC	0.2369	0.0864	(0.0561, 0.4178)	0.0130
Cytokine				
IL15	0.2712	0.1204	(0.0192, 0.5231)	0.0363
^b^VEGF-A	0.4755	0.2164	(0.0226, 0.9284)	0.0406
Obesity				
^b^BDNF	1.3501	0.5397	(0.2205, 2.4797)	0.0217

a *p*-values from the GLM were adjusted for baseline values of each blood analyte.

b Indicates natural logarithm transformation prior to analysis for values of serum analytes that did not follow a normal distribution.

c Indicates significance following Bonferroni-Holm correction for multiple comparisons.

**FIGURE 3:**
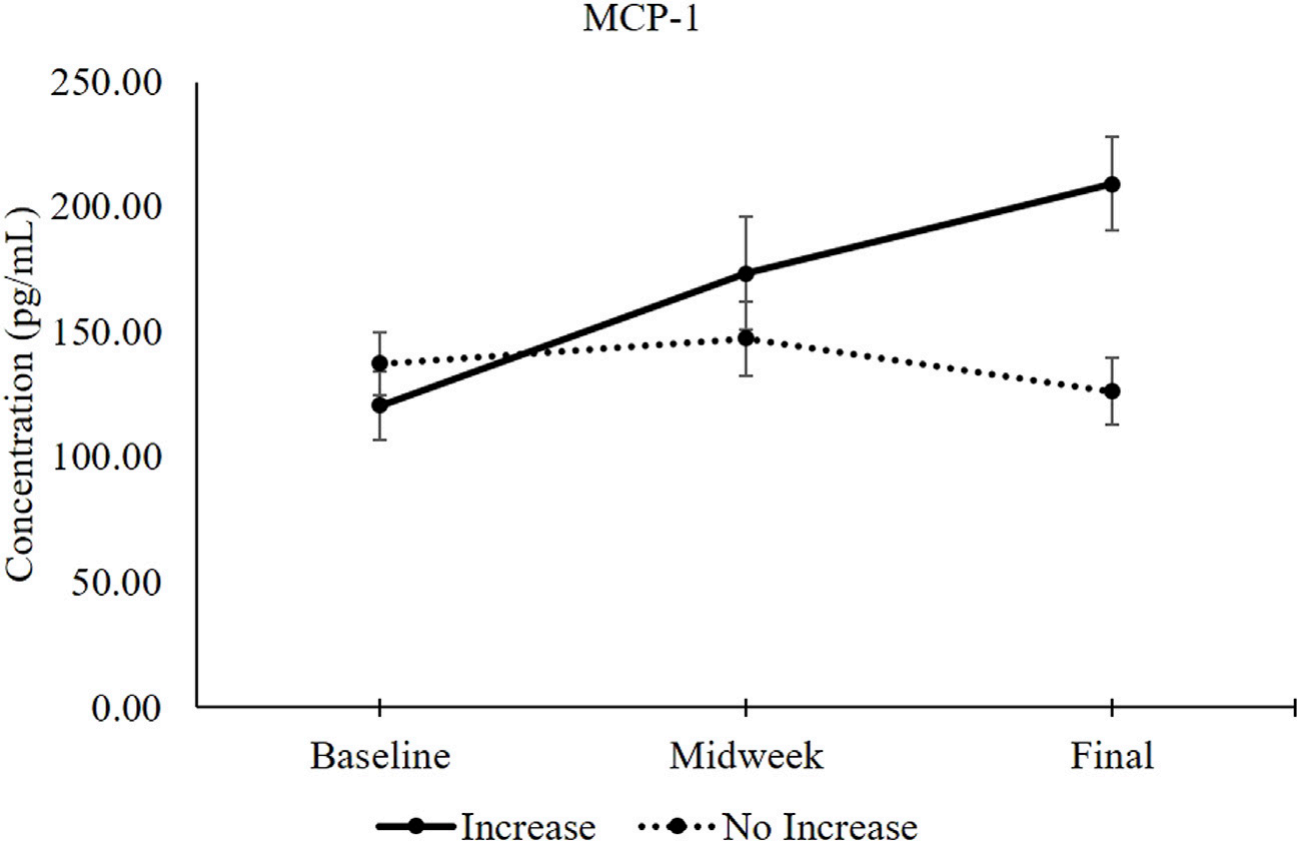
MCP-1 serum levels between participants with increased General Fatigue (*n* = 13) and without increased General Fatigue (*n* = 9). The solid black line represents MCP-1 serum levels (mean ±1 SEM) in participants whose General Fatigue score increased between baseline and the study endpoint. The dotted black line represents MCP-1 serum levels in participants whose General Fatigue did not increase (Table 5). Independent samples *t*-test comparisons revealed 2-tailed *p*-values of 0.404 at baseline, 0.345 at midweek and 0.004 at the study endpoint.

Figure 3 provides a graphical comparison between levels of MCP-1 within participants who experienced increased levels of General Fatigue versus those who reported no increase. Baseline MCP-1 levels did not differ between persons who would later become fatigued versus those who would not. This figure also illustrates that aviators endorsing General Fatigue experienced a 44% increase in MCP-1 serum levels by the midweek data collection. At the study endpoint, their MCP-1 levels had increased to 74% above baseline.

## Discussion

This study’s objective was to characterize physiologic responses to repetitive sorties within a cohort of USAF T-6A Texan II instructor pilots. Primary outcomes included serial measurements and quantification of serum proteins produced and released in response to inflammatory processes, vascular and angiogenic stimuli, as well as cognitive and physical challenges to the central nervous system. Secondary outcomes included simultaneous measures of fatigue, general blood chemistry and urine specific gravity. We found that baseline measures of serum proteins were comparable to currently available reference values (Biancotto et al., 2013; Wu et al., 2017). By the conclusion of the second day of the flying schedule, serum levels of multiple analytes diverged from baseline values, with 20 becoming significantly different by the fourth day of flying.

We also found that the study cohort as a whole endorsed an increase of General Fatigue by the conclusion of the study period (Table 5). That overall increase was driven by an escalation of General Fatigue in 13 out of 22 study participants, whose reported levels were similar to values observed within persons characterized as “unwell” by the Centers for Disease Control and Prevention (Lin et al., 2009). Those same 13 participants also reported increased Mental Fatigue, Reduced Motivation, and Reduced Activity levels (Table 5).

We considered whether dehydration, an ever-present physiologic stressor within the tactical aviation environment, could have been present and contributed to our findings. Urine specific gravity, measured at the same time venous blood was collected, suggests that the aviators met criteria for dehydration, defined by specific gravity measures ≥1.020 (Popowski et al., 2001), during baseline data collection, and were “hypohydrated,” defined by specific gravity measures ≥1.013 but ≤1.020 (Brescon et al., 2018), during the remainder of the study period. As an additional measure, we assessed whether hydration status was accompanied by plasma loss and hemoconcentration, manifest as increased red blood cell mass per unit of plasma (Watanabe et al., 2020). This would have been reflected as an increased hematocrit (Hct) level within venous blood samples measured at midweek and endpoint data collection (Table 3). However, Hct levels were within the normative range (42.6%–47.1%) (Gagnon et al., 1994) as was plasma volume (2.98–3.19 L) (Yiengst and Shock, 1962), and neither changed across the data collection time points. Collectively, measures of urine specific gravity and Hct suggests that dehydration was unlikely to be among the potential physiologic stressors contributing to the increased levels of fatigue and serum analyte changes we observed (Tables 4–7).

Serum analyte profiles that emerged within the study cohort across their flying schedule offer novel insight into physiologic responses to sorties. Three of nine analytes measured with the “Proinflammatory Plate” were different between baseline and the final data collection time point (Table 6). Of those three, IL-8 is among the better-described proinflammatory interleukins that attract neutrophils to a site of injury (Kany et al., 2019). In contrast, IL-4 and IL-10 are generally considered as anti-inflammatory interleukins (Chatterjee et al., 2014). Despite the rise of IL-4 across the study period, overall levels of both IL-4 and IL-10 were lower than those found within healthy persons, and comparable to levels observed in patients reporting chronic pain and inflammation (Üçeyler et al., 2006). During conditions of chronic inflammation, reductions in IL-4 and IL-10 are thought to suppress proinflammatory activity and subsequently minimize tissue damage (Saraiva and O'Garra, 2010; Ouyang et al., 2011). While assessment of self-reported pain or measures of chronic inflammation were beyond the intention and scope of this study, tactical aviators frequently experience musculoskeletal duress (Sovelius et al., 2008; Thoolen and van den Oord, 2015), which progresses to degenerative inflammatory processes within cervical intervertebral disks (Hämäläinen et al., 1993).

We also found six of nine analytes measured with the “Chemokine Plate” increased between baseline and endpoint data collection (Table 6). Of these, MCP-1 influences movement of monocytes out of the bloodstream, across the endothelium and into the tissues to engage in immunologic surveillance and inflammatory responses (Deshmane et al., 2009). Similar in function is MCP-4, a chemoattractant for monocytes as well as eosinophils (Garcia-Zepeda et al., 1996). Eotaxin-3 also functions as a chemoattractant for eosinophils and contributes to inflammatory processes within the airway, typically following exposure to an antigen (Tateno et al., 2001; Yamada et al., 2019). Macrophage inflammatory protein alpha (MIP-1α), which also became elevated, is an inflammatory chemokine produced in response to inflammatory or infectious processes. Similar to that is macrophage inflammatory protein beta (MIP-1β), which also increased. MIP-1β recruits leukocytes into injured tissues to perpetuate inflammatory processes (Dumas et al., 2016). TARC, the last of the analytes to be increased on the Chemokine Plate, is associated with type 2 immune responses (Catherine and Roufosse, 2021). Collectively, these changes correspond with the presence of both chronic and acute inflammatory processes.

Serum analyses also revealed that five of nine analytes measured with the “Cytokine Plate” increased between baseline and the final data collection time point (Table 6). Those cytokines share common functions related to the onset of inflammatory processes and protective immune responses (Perera et al., 2012). Among those was IL-7. This component of lymphocyte development and T cell homeostasis (Lundström et al., 2012) also contributes to proinflammatory processes (van Roon et al., 2005). IL-12, which also increased, is considered to be a proinflammatory cytokine influencing T-cell and natural killer-cell responses (Trinchieri, 2003). Consistent with the theme of increased levels of cytokines mediating immune responsiveness and inflammation, both IL-15 and IL-17A increased. In addition, VEGF-A, an angiogenic promoter typically released in response to inflammatory conditions, glucose deficiency, or hypoxia (Regenfuss and Cursiefen, 2010) increased 2.7 fold between baseline levels and those measured at the study endpoint. An alternative consideration, relevant to this study’s participants, is that VEGF-A increased in response to hyperoxia (Barratt et al., 2018; Hadanny and Efrati, 2020).

By the fourth day of the flying schedule, which was the last day of data collection, two out of six analytes measured on the “Angiogenesis Plate” were reduced below baseline levels (Table 6). These were VEGF-D and TIE-2, which are involved with angiogenesis, growth, and remodeling of blood and lymphatic vessels (Sudhadevi et al., 2021). Reductions in these serum analytes are rarely encountered, other than from exposure to hyperoxia. That physiologic stressor to lung tissue evokes an increase of sphingosine-1-phosphate which subsequently reduces multiple angiogenic factors, including VEGFs and TIE-2 (Sudhadevi et al., 2021).

Of the four analytes measured with the “Vascular Plate,” only VCAM-1 changed across the study period. Vascular Cell Adhesion Molecule-1 levels are known to increase in the presence of pro-inflammatory cytokines, and/or increased blood glucose, as well as from shearing stress (Kong et al., 2018). However, we found that VCAM-1 was reduced from baseline values by 38%. The increased levels of VEGF-A (Table 6) and other proinflammatory proteins may have stimulated production of Angiopoietin-1 (ANG-1) within vascular smooth muscle cells. Recently, ANG-1 has been shown to counteract VEGF-induced inflammatory processes by reducing leukocyte adhesion through suppression of VCAM-1 and other cell adhesion proteins (Kim et al., 2001). However, whether ANG-1 induced suppression of VCAM-1 in our study cohort is only speculative, as we did not measure ANG-1 during this study.

We also observed three out of five analytes measured on the obesity plate changed significantly between baseline and the study endpoint. Of these, BDNF, a neurotrophin thought to be involved with memory formation of environmental experiences, was significantly elevated. Prior studies have shown that exercise and cognitive training lead to increased BDNF levels, which was associated with enhanced performance during memory tasks (Miranda et al., 2019). BDNF is also increased in human serum following exposure to hypergravity (Bae et al, 2018), as well as within the lungs of mice exposed to hypergravity (Antonelli et al., 2002).

It is feasible that the hypergravity (+Gz) maneuvers routinely experienced by each of the aviators contributed to the increased BDNF levels (Bae et al, 2018) that first emerged during the midweek (Tuesday) portion of this study. Another consideration is that instructor pilots experience continual cognitive challenges during in-flight training of their students. As BDNF levels were associated with increased General Fatigue (Table 7), we suspect that both the sortie-related exposures to +Gz and cognitive challenges associated with student pilot training contributed to the substantial elevations in serum BDNF that emerged.

Serum samples measured with the obesity plate also revealed reductions in both leptin and glucagon across the study period. Circulatory levels of leptin reflect fat mass, influence food intake, and can lower blood glucose levels (Frühbeck et al., 1998; D'Souza et al., 2017). Leptin levels are decreased during fasting or can substantially increase following a single day of over eating (Tatti et al., 2001). Leptin levels, measured throughout the study, were somewhat lower than values of 9.0 ± 0.83 ng/ml reported in males with a mean age of 41.1 ± 2.49 years and BMI of 25.8 ± 0.43 (Isidori et al., 2000), but similar to values of 6.50 ng/ml found in males aged 30–39 years (Gijon-Conde et al., 2015).

Glucagon levels decreased across the study period. Reduced blood glucose stimulates increased glucagon levels, which triggers the liver to initiate glucose production that maintains adequate blood levels (Taborsky, 2010). Consistent with that function, glucagon levels are increased during fasting and reduced after eating. We found baseline glucagon serum levels, measured on Sunday afternoon, were almost two-fold higher than levels measured during the weekday portion of the study period. Sleep and circadian cycles influence leptin (Spiegel et al, 2004) while food intake influences glucagon (Frühbeck et al., 1998). As neither sleep duration nor food intake were controlled during this study, we cannot speculate upon whether the decreased levels we observed reflect changes in sleep, diet or both that may have occurred during this week-long study.

Causative factors contributing to the changes in blood serum analytes and increased General Fatigue could not be identified by this study’s design. Nonetheless, the progressive increase in proinflammatory cytokines that emerged across the entire cohort throughout the study period informed our hypotheses of potential factors. We believe that exposures occurring during each sortie, including frequency, duration and +Gz, contributed to the increased BDNF and proinflammatory cytokines that we observed. Findings of increased serum neurotrophin levels following centrifuge studies concur with our hypothesis (Bae et al, 2018). Continuous inspiration of dry hyperoxic gas, similar to that provided to each aviator through their plane’s life support system, can also evoke release of proinflammatory cytokines (Huang et al., 2016). Increased cognitive workload/stress, which has also been shown to promote increased serum levels of proinflammatory cytokines (Marsland et al., 2017), is another consideration. Collectively we propose that multiple physical challenges, inherent to tactical aviation, contributed to the observed changes in blood serum analytes.

An individual’s physiological response to sortie-induced physical challenges can be mediated by multiple factors and behaviors. These include physical condition, presence of chronic inflammatory processes, sleep, diet, exercise, and activities of daily living. Individual differences in those traits and behaviors may have contributed towards the onset of fatigue reported by some aviators. For example, multiple participants may have been afflicted with chronic muscle and bone inflammation, which could be inferred by reduced levels of IL-4 and IL-10 (Table 6). Dietary habits, perturbed sleep or other behaviors/traits that were not monitored in this study may have also conferred vulnerability towards the fatigue-inducing effects of proinflammatory cytokines (Raison et al., 2006). Future studies establishing whether those conditions and behaviors did indeed exist and may have differed between aviators who did or did not become fatigued, will provide rationale for interventions seeking to enhance resiliency against sortie-related physical challenges.

### Strengths and Limitations

We believe this may be the first study to obtain serial serum measurements of cytokines, chemokines, angiogenic proteins, and corresponding levels of fatigue across a 5-day period in a cohort of military aviators. Among the limitations of this field study was the requirement that all data collection be performed without interfering with each participant’s daily duty requirements, time schedule, mealtimes, and rest periods. Therefore, data collection occurred at the conclusion of each participant’s workday schedule, typically between 3:00 PM-5:00 PM. It is feasible that meal and sleep schedules may have influenced leptin and glucagon levels. However, this study’s primary outcomes, including serum levels of cytokines, chemokines, and BDNF, are more reflective of immune system activity rather than dietary or sleep schedules (Wu et al., 2017). Thus, we do not believe that this approach negatively affected our study protocol, data collection paradigm or study outcomes.

The inability to obtain the actual number and duration of each +Gz maneuver that study participants experienced during their sorties confounded our quantification of actual time spent at +Gz. That information is captured only by a recording device within each plane. Obtaining those data would have required that the plane be removed from service and the data downloaded, processed, and analyzed, which was not an option. This prevented rigorous comparisons between +Gz with blood serum analytes and levels of fatigue. Nonetheless, this study’s findings do suggest potential relationships exist between sortie frequency, duration, and cumulative +Gz exposure with onset of fatigue. Future studies with controlled and measurable exposures are warranted to precisely define the relationship between the physiologic stressors of successive sorties with subsequent blood serum proinflammatory cytokine levels and fatigue onset.

Another consideration was the time span between data collection points. Prior to designing this study, we performed preliminary work in which serial blood collections occurred prior to each sortie, and then immediately afterwards, as well as every 8–12 h for the next 48 h. Those data revealed that changes in blood serum analytes (of interest) did not begin to emerge until approximately eight to 12 h after each sortie. Blood serum levels then remained elevated for several hours, and only began to gradually decline if no additional sorties occurred. In contrast, if follow-on sorties did occur, the analytes remained elevated, with many achieving levels that were markedly greater than those emerging after only a single sortie. Therefore, we believe that data collected on a Tuesday afternoon (midweek) provided insight into physical challenges occurring the day before (Monday) while data collected at the study endpoint (Thursday) provided insight into cumulative activities occurring during the prior day (Wednesday) and perhaps Tuesday (see Figure 1). Although not an optimal design with which to establish cause and effect, it was considered too disruptive to collect data prior to each morning’s briefings and preparations for syllabus-driven training sorties. In addition, this schedule is an accurate representation of an instructor pilot’s duty day, suggesting a high ecological validity of this approach.

Serial measurements of serum cytokine levels typically occur in persons experiencing pathological processes, with those levels compared against non-diseased matched controls (Wu et al., 2017). This has led to a lack of publications describing day-to-day stability in serum proinflammatory cytokine levels and other analytes that we selected to study. However, we did identify two studies that obtained serial measurements of many of the same analytes that we focused upon, measured in healthy participants who were of similar age to our study participants. Those studies found that serum cytokine levels remained stable across 1 week (Biancotto et al., 2013) and across 14 weeks (Wu et al., 2017), in persons who were not engaged in high duress physical activities. Together, those study findings support our speculation that the changes in serum analyte profiles we observed occurred in response to specific stimuli rather than the result of daily variation.

Statistical analyses revealed that serum levels of 20 out of 42 analytes differed between baseline and the study endpoint. To inform our decision of whether Bonferroni-Holm corrections were warranted, we identified recent studies of a similar sample size, n = 20 (Kerstein et al, 2017) and larger n = 51 (Wunderlich et. al., 2017), which measured blood serum markers across multiple time points, using similar technologies as employed in this study. Neither study applied Bonferroni corrections. Nonetheless, we felt that controlling for Type I error could enhance the rigor of our analyses and defensibility of findings. After applying Bonferroni-Holm corrections, we found that 13 out of 20 serum analytes remained significantly different between baseline and the study endpoint. As application of Bonferroni corrections in a study such as this could obscure pertinent findings (Armstrong, 2014), we chose to present all significant findings and annotate those that remained significant following Bonferroni-Holm corrections. We consider this level of transparency to be a strength rather than a limitation of this study.

We do not believe that this study’s limitations impact the significance of our findings that T-6A Texan II instructor pilots experienced increased serum levels of proinflammatory cytokines across their week-long flying schedule, and that General Fatigue and Reduced Motivation levels increased in parallel with those cytokines. Those observations, derived from a unique cohort of USAF active duty and reservist aviators, concur with prior experimental and medical patient-based studies (Raison et al., 2010; Dantzer et al., 2014; Sofroniew, 2014; Karshikoff et al., 2017; Zielinski et al., 2019; Lasselin et al., 2020) to suggest that mechanisms other than sleep loss contributed to the onset of fatigue within the aviators. This study’s findings also suggest the need to determine why some instructor pilots were more resilient than others and less fatigued following similar exposures of musculoskeletal duress, +Gz, hyperoxia, increased work of breathing, acceleration atelectasis, and other physical and cognitive challenges inherent to tactical aviation. This knowledge will create new opportunities to develop additional strategies aimed at mitigating the burgeoning threats imposed by aviator fatigue.

## Data Availability

The datasets presented in this article are not readily available because the data are subject to third party restrictions. The data are not openly available due to privacy or ethical restrictions. Data supporting the findings of this study may be available from the Department of the Navy upon reasonable request submitted to the corresponding author. Requests to access the datasets should be directed to mjd6@case.edu.
